# The Treatment of Measles a "Mere Matter of Routine"

**Published:** 1898-01-15

**Authors:** 


### The Hospital

A Journal of

### The Medical Sciences and Hospital Administration.

Vol. XXIII.—No. 590.] Edited by Sir Henry Burdett, K.C.B., and Solomon C. Smith, M.D., M.R.C.P. [Jan. 15, 1898.

### The Treatment of Measles a "Mere Matter of Routine."

We regret to observe a tendency among medical officers of health to take up positions on medical subjects more or less antagonistic to those generally acceptable to their professional brethren. Perhaps this is to some extent the result of their occupying an official position—perhaps it is partly the result of the growing specialisation of hygiene as a subject apart from general medicine; but, in any case, it is to be regretted. The point to which we would now direct attention is the proposed employment of nurses for undertaking the "routine treatment" of ordinary cases of measles and whooping cough.

The more familiarised we become with the custom of having a nurse as a helper in dealing with disease the more plain does it appear that, if both doctor and nurse are to be of full utility, the functions of each must be kept separate and distinct. By mutual agreement it is now generally recognised that the duty of the nurse is to carry out the instructions of the medical man, and that the nurse is stepping out of her province altogether if she attempts to undertake the treatment of disease—for which, indeed, she has received neither training nor qualification.

The General Medical Council also has recently issued its fiat on the subject of unqualified practice, warning the profession against the employment of unqualified persons as assistants. This it has done on the broad ground that unqualified practice is a danger to the public, and it is now thoroughly understood that the practice of medicine, the doing of anything which requires the exercise of "professional discretion or skill," by unqualified persons, is detrimental to public interests.

On looking through the last number of *Public Health* we find a series of pronouncements on the notification of measles taken from the annual reports of medical officers of health in various parts of the country. Almost without exception they insist on the gravity of the disease, its hidden dangers, and its high mortality. Nor need we be surprised at this when we bear in mind that, taking an average of years, it is by far the most fatal zymotic disease that we have among us. Dr. Kenwood lately pointed out that measles caused more deaths of children under five years of age than were caused by small-pox, scarlet fever, diphtheria, and typhoid fever all together. In view of this high mortality, and of the admitted fact that parents comparatively seldom call in medical advice for the treatment of the disease, one might have expected that the very first step taken by medical officers of health would be to insist that measles should be seriously treated, and that the little patients should be provided with proper medical attendance. This, however, is not the view that commends itself to the official mind. In the midst of all this loss of life from measles and whooping cough, Dr. Bond, of Gloucester, writes as follows: "There is only one form of action which in my opinion, would be of any material use in helping to lessen the severity of these two diseases, and that is the employment by the sanitary authority of a well-trained nurse to visit every house in any district in which either of them might be prevalent for the purpose of teaching the mothers how *to treat* the children so as to avoid prejudicial results. . . . Whenever a case of either affection made its appearance in any locality she would be appealed to as a matter of course, and by taking up her abode for a time in the affected locality she would be able to *do all that is practicable to control the ravages of these diseases, the medical treatment of which is in ordinary cases a mere matter of routine, which any intelligent woman could with a little training undertake.*" The italics, of course, are ours.

Now it appears to us a very serious matter that a medical man occupying an official position should advocate such an extension of unqualified practice as is here suggested, just at the very time that the General Medical Council has pronounced so definitely against unqualified practice of all kinds. It is idle to say, as Dr. Bond does, that what he proposes is not a question of "putting an unqualified woman in the place of a qualified practitioner," since "in the large majority of cases a doctor is not called in at all." This is no excuse. The very point is that a doctor ought to be called in. We do not claim the treatment of these measles and whooping-cough cases as a perquisite (!) for the medical profession. What we do claim is that these poor children should have proper treatment by qualified medical men when suffering from ailments which are now known to be among the most treacherous and fatal of the diseases of infancy. Dr. Bond says that the "intelligent nurse" whom he would trust "to control the ravages of these diseases, the medical treatment of which is in ordinary cases a mere matter of routine," would be able to advise the parents "when danger was really imminent and when the aid of a skilled adviser was necessary." But it is this putting off the sending for the doctor till danger is "really imminent" which is the very root of the matter. If sanitary authorities want to diminish the loss of life from measles, and do not want to establish hospitals for their reception, let them by all means send a doctor and a nurse. Let them insure that the patients have skilled treatment from the beginning, but do not let them call into being an army of unqualified medical advisers to undertake the "routine treatment" (!) of such serious diseases.

